# Supplementary figures and images for: Multi-Level Multi-Growth Models: New opportunities for addressing developmental theory using advanced longitudinal designs with planned missingness
Source: Dev Cogn Neurosci. 2021 Aug 8;51:101001. doi: 10.1016/j.dcn.2021.101001 (PMC8363832; doi:10.1016/j.dcn.2021.101001)

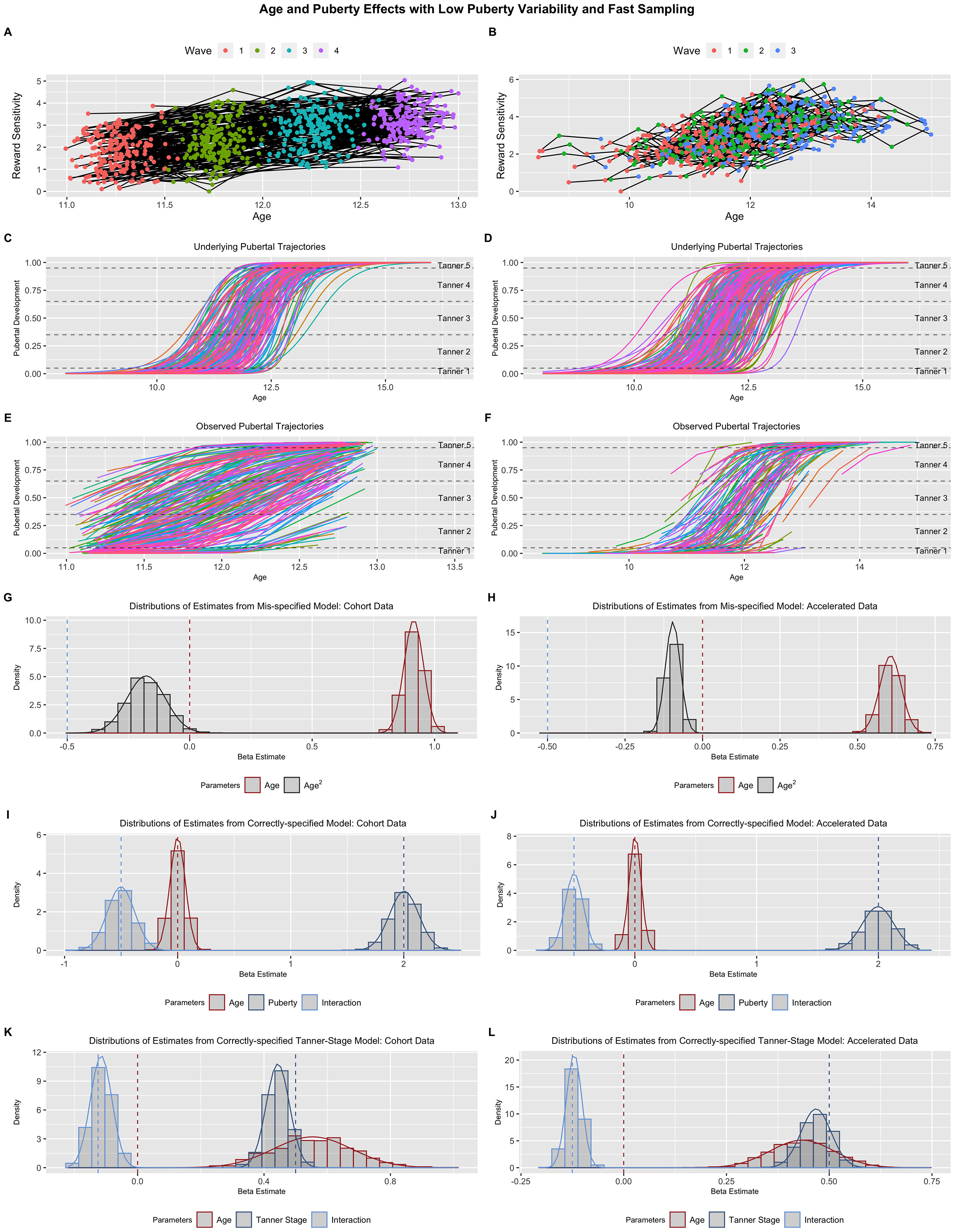

Supplement: Supplementary file 1 [file mmc1.zip › mmc6.jpg]

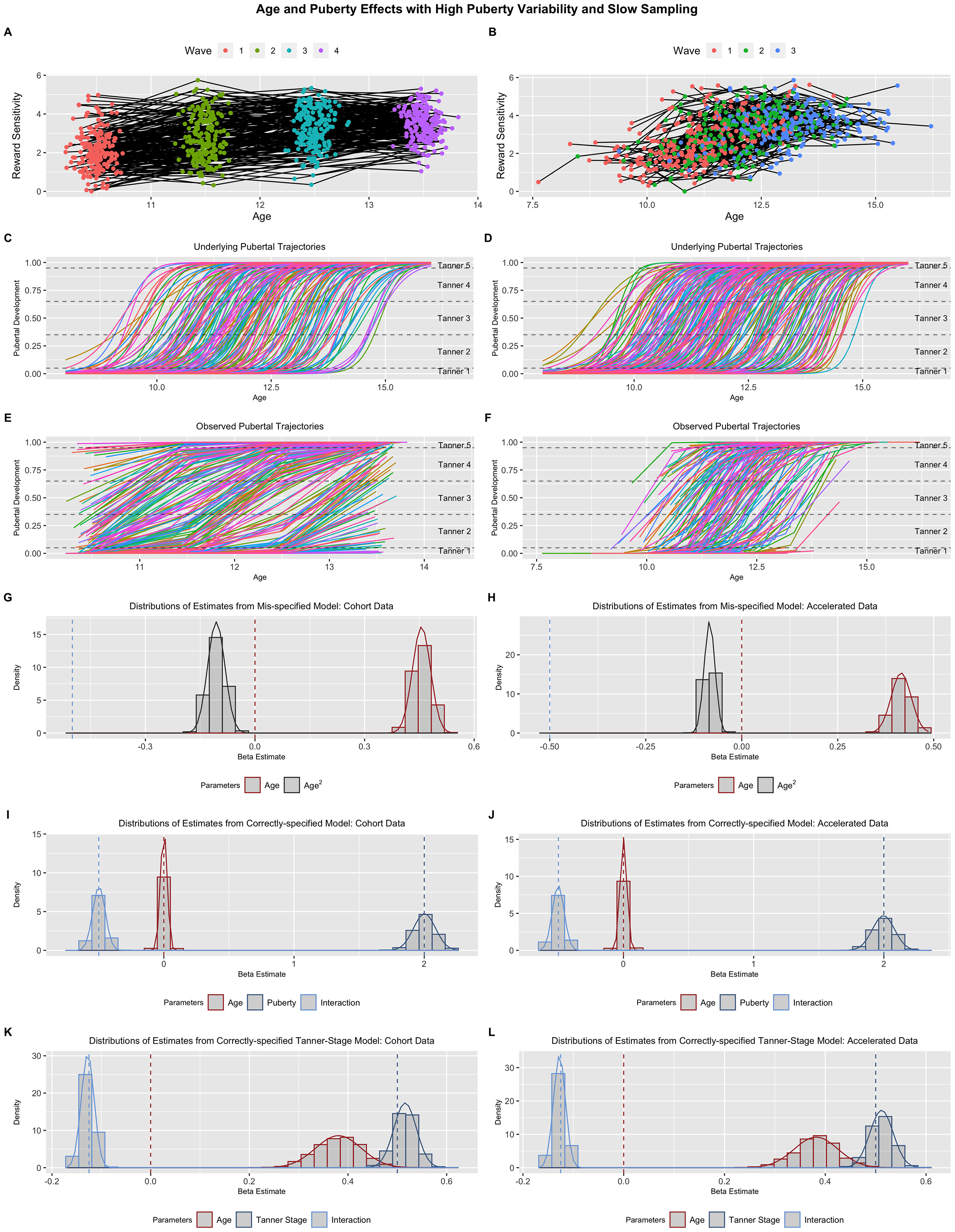

Supplement: Supplementary file 1 [file mmc1.zip › mmc7.jpg]

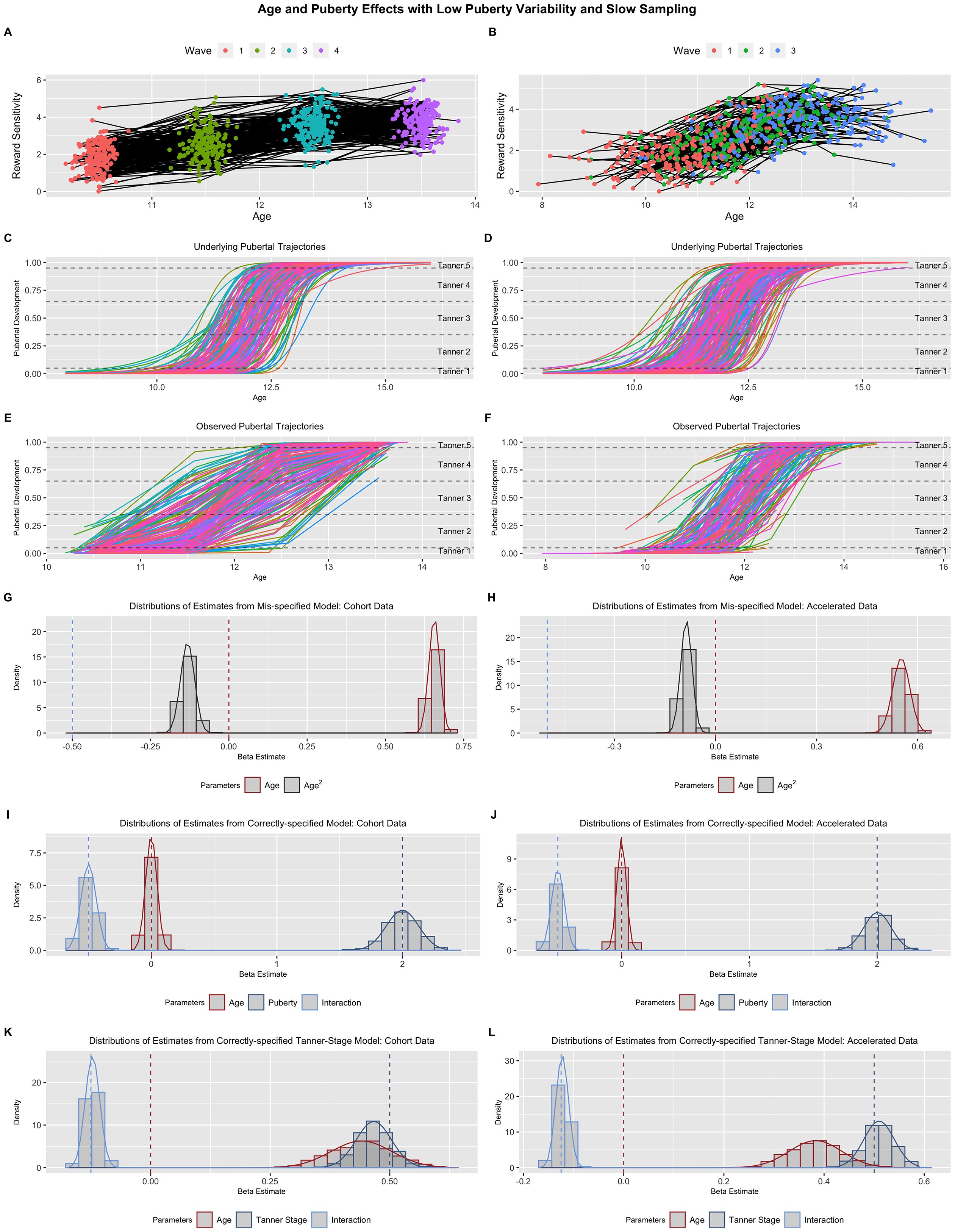

Supplement: Supplementary file 1 [file mmc1.zip › mmc8.jpg]

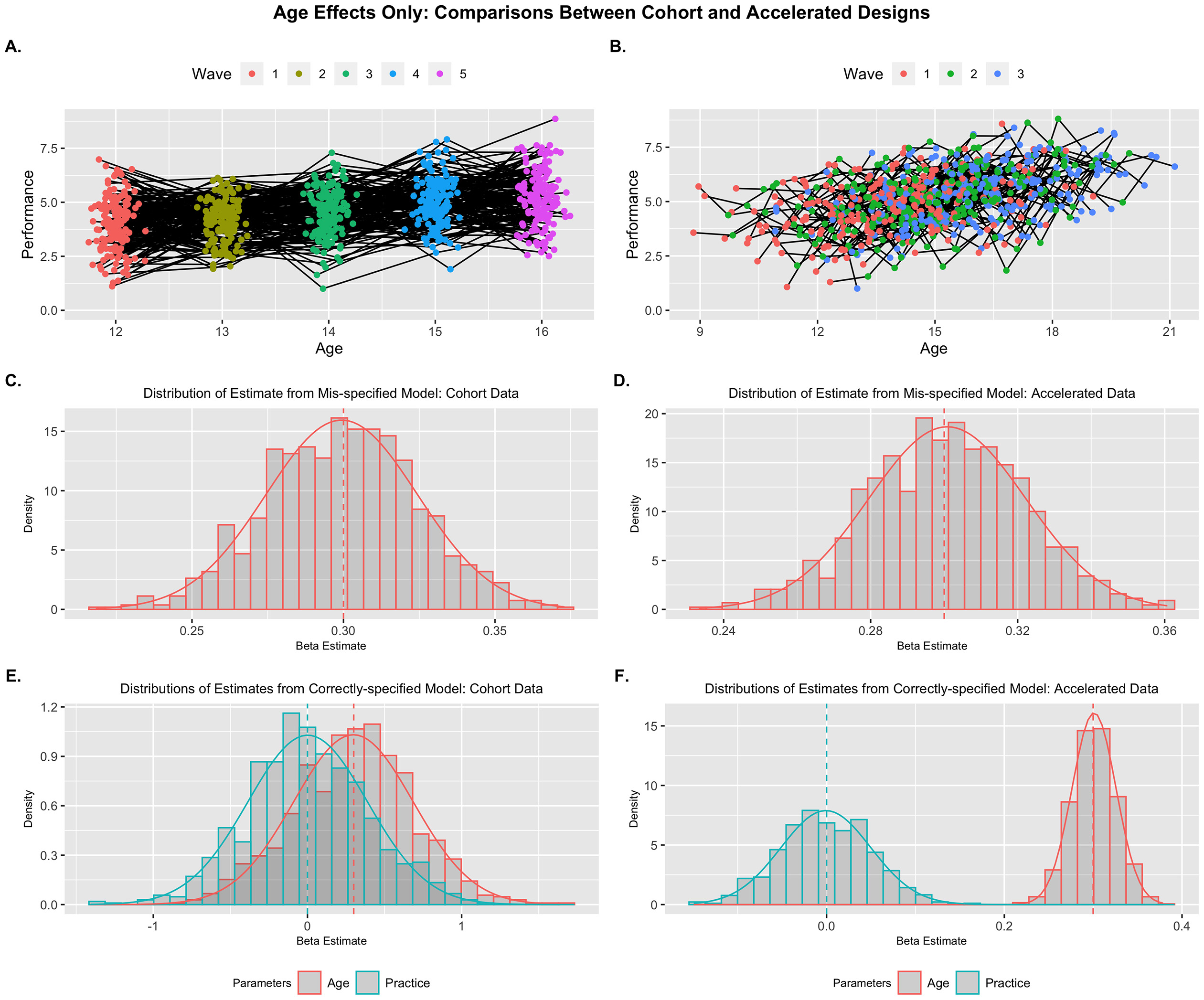

Supplement: Supplementary file 1 [file mmc1.zip › mmc1.jpg]

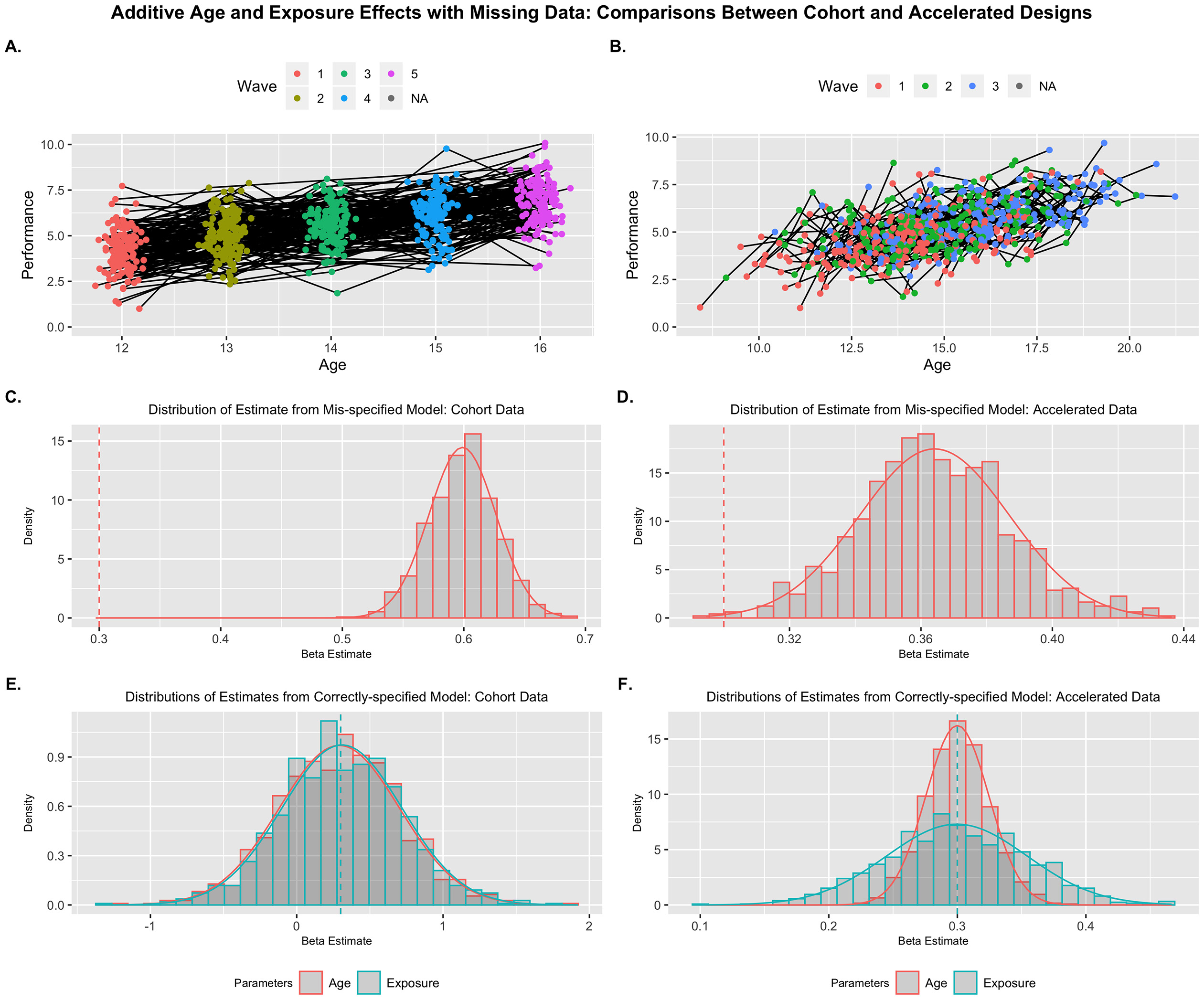

Supplement: Supplementary file 1 [file mmc1.zip › mmc2.jpg]

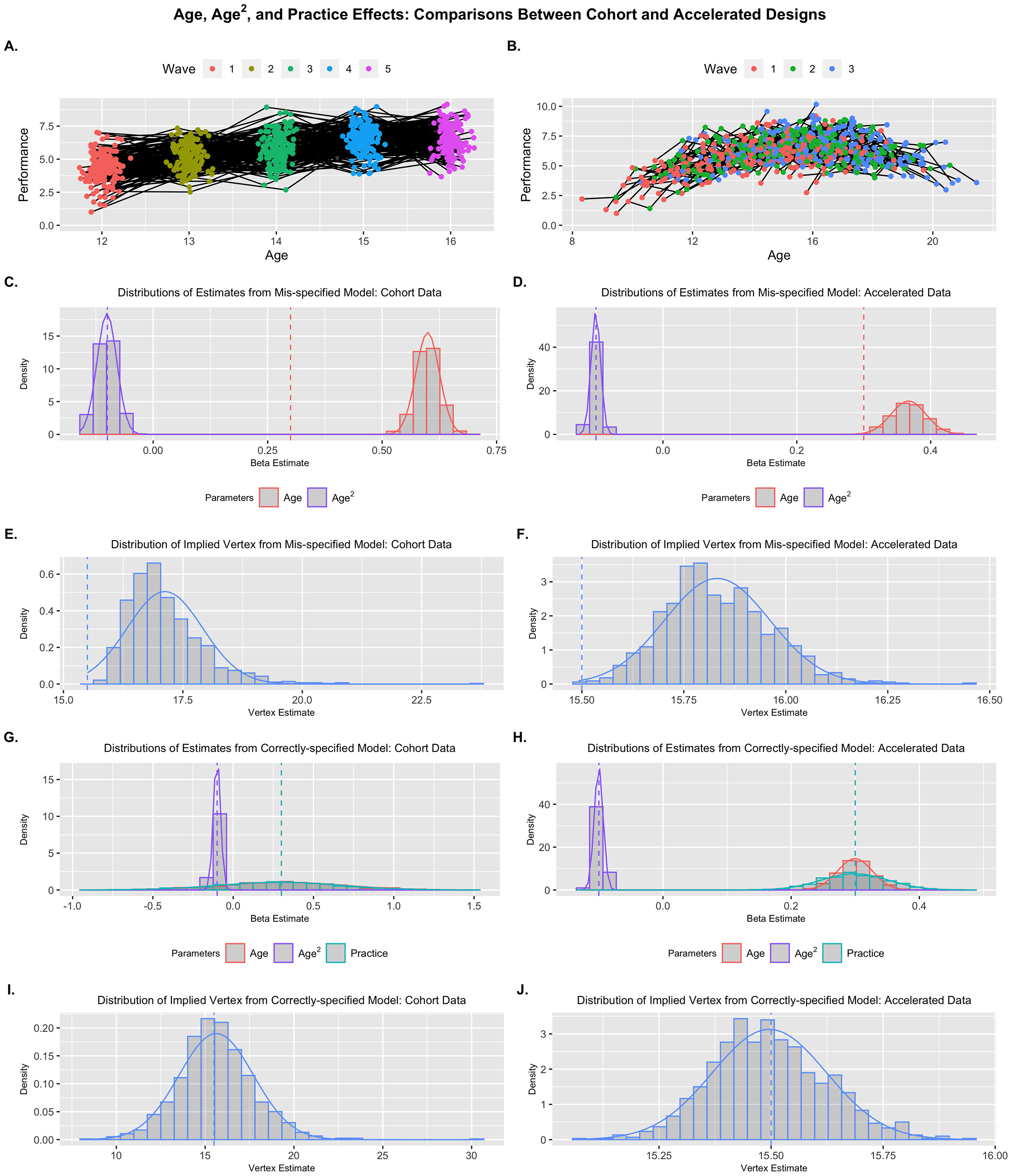

Supplement: Supplementary file 1 [file mmc1.zip › mmc3.jpg]

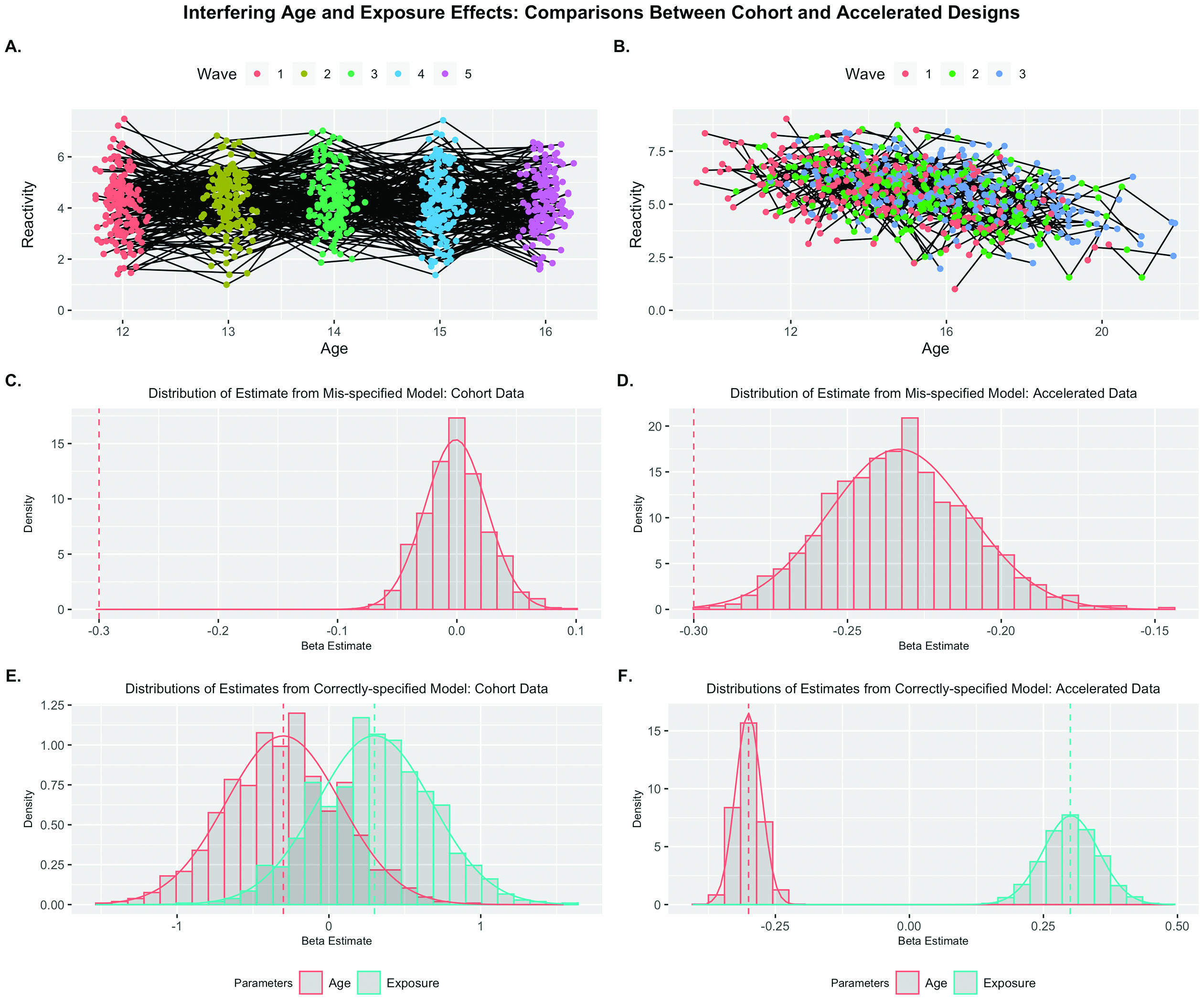

Supplement: Supplementary file 1 [file mmc1.zip › mmc4.jpg]

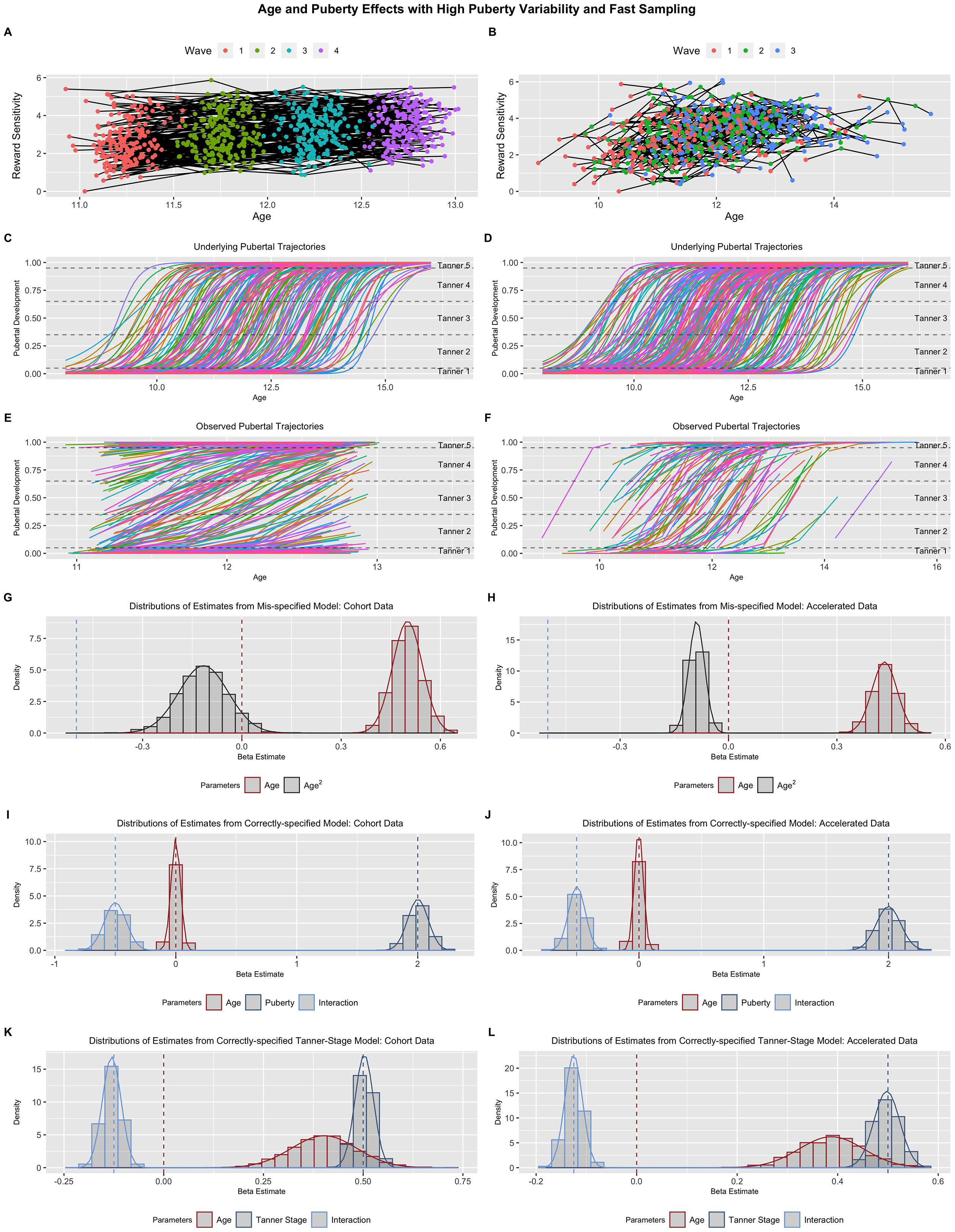

Supplement: Supplementary file 1 [file mmc1.zip › mmc5.jpg]
